# Diurnal variability of glucose tetrasaccharide (Glc_4_) excretion in patients with glycogen storage disease type III

**DOI:** 10.1002/jmd2.12181

**Published:** 2020-11-03

**Authors:** Sarah P. Young, Aleena Khan, Ela Stefanescu, Andrea M. Seifts, Ghada Hijazi, Stephanie Austin, Priya S. Kishnani

**Affiliations:** ^1^ Division of Medical Genetics, Department of Pediatrics Duke University School of Medicine Durham North Carolina USA; ^2^ Duke University Health System Biochemical Genetics Laboratory Durham North Carolina USA

**Keywords:** 24‐hour urine, biomarker, Glc_4_, glucose tetrasaccharide, glycogen storage disease type III, Hex_4_, uncooked cornstarch

## Abstract

**Aim:**

The urinary glucose tetrasaccharide, Glcα1‐6Glcα1‐4Glcα1‐4Glc (Glc_4_), is a glycogen limit dextrin that is elevated in patients with glycogen storage disease (GSD) type III. We evaluated the potential of uncooked cornstarch therapy to interfere with Glc_4_ monitoring, by measuring the diurnal variability of Glc_4_ excretion in patients with GSD III.

**Methods:**

Voids were collected at home over 24 hours, stored at 4°C and frozen within 48 hours. Glc_4_ was analyzed using liquid chromatography‐tandem mass spectrometry and normalized to creatinine.

**Results:**

Subjects with GSD III (median age: 13.5 years, range: 3.7‐62; n = 18) completed one or more 24‐hour urine collection, and 28/36 collections were accepted for analysis. Glc_4_ was elevated in 16/18 subjects (median: 13 mmol/mol creatinine, range: 2‐75, reference range: <3). In collections with elevated Glc_4_ (23/28), two‐thirds (15/23) had low diurnal variability in Glc_4_ excretion (coefficient of variation [CV%] <25). The diurnal variability was significantly correlated with the Glc_4_ concentration (Pearson *R* = .644, *P* < .05), but not with the dose of uncooked cornstarch. High intraday variability (>25%) was not consistently observed in repeat collections by the same subject.

**Conclusions:**

The extent and variability of Glc_4_ excretion relative to creatinine was not correlated with cornstarch dose. A majority of collections showed low variability over 24 hours. These findings support the use of single time‐point collections to evaluate Glc_4_ in patients with GSD III treated with cornstarch. However, repeat sampling over short time‐periods will provide the most accurate assessment of Glc_4_ excretion, as intraday variability may be increased in patients with high Glc_4_ excretion.

**Abbreviations**
ALT: alanine transaminase
AST: aspartate transaminase
CK: creatine kinase
CN: creatinine
GDE: glycogen debranching enzyme
Glc_4_: glucose tetrasaccharide, Glcα1‐6Glcα1‐4Glcα1‐4Glc
GSD: glycogen storage disease
HCC: hepatocellular carcinoma
IS: internal standard

**Synopsis**

The diurnal variability of Glc_4_ excretion is low for a majority of patients with glycogen storage disease III and is not correlated with cornstarch dose.

## 1 INTRODUCTION

Glycogen storage disease type III (GSDIII, MIM #232400) is an autosomal recessive disorder caused by a deficiency of glycogen debranching enzyme (GDE, EC 3.2.1.33, EC 2.4.1.25) encoded by *AGL* (MIM #610860). GDE is a cytosolic enzyme that works in combination with glycogen phosphorylase to release glucose from glycogen for energy metabolism. GDE deficiency disrupts glucose homeostasis, and results in an accumulation of abnormally structured glycogen enriched in α‐1‐6 branch points.^1^ The clinical manifestations are variable and patients are classified as GSD IIIa, characterized by liver, heart, and muscle involvement, or GSD IIIb in which the liver is predominantly affected.^2^ GSD III often presents in infancy or childhood with hepatomegaly and hypoglycemia due to liver disease. Ketosis, hyperlipidemia, and growth retardation are common.^3^ Skeletal muscle weakness slowly progresses in GSD IIIa, becoming more prominent in the third to fourth decades of life. Liver disease becomes less apparent in adolescence and adulthood, and is associated with a decrease in liver size and serum aminotransferase levels.^4^ However, long‐term hepatic complications have been reported including liver fibrosis, cirrhosis, adenomas, and hepatocellular carcinoma.^5^, ^6^

In addition to the general management of disease manifestations, treatment includes dietary therapy using uncooked cornstarch to minimize hypoglycemic events, and a high protein diet as an alternative energy source.^7^, ^8^ As new therapies are being investigated,^8^, ^9^, ^10^ biomarkers are needed to determine the clinical severity and monitor disease progression. The glucose tetrasaccharide, Glcα1‐6Glcα1‐4Glcα1‐4Glc (Glc_4_) is a glycogen limit dextrin produced by circulatory amylases and neutral α1‐4‐glycosidases.^11^ It is elevated in conditions associated with increased glycogen accumulation and/or release of glycogen from damaged tissues.^12^, ^13^, ^14^, ^15^, ^16^ Urinary Glc_4_ is an established biomarker in patients with Pompe disease, correlating with skeletal muscle glycogen and disease status in these patients.^17^, ^18^, ^19^ Glc_4_ is also elevated in GSD III,^16^, ^20^, ^21^, ^22^, ^23^ and has potential as a biomarker in this disorder. Glc_4_ is usually measured in randomly collected voids (spot urines) for the convenience of patients and clinical personnel. However, it has not been determined whether Glc_4_ measurements in spot urines represent excretion over a 24‐hour period. Previous studies have suggested that ingestion of starch or glycogen may increase Glc_4_ excretion,^14^ and in GSD III this could be a particular concern because of cornstarch therapy. We evaluated the diurnal variability in Glc_4_ excretion, to investigate whether the degree of elevation and variability in Glc_4_ excretion correlated with cornstarch therapy in patients with GSD III.

### 1.1 Materials

Acquity UPLC BEH amide 2.1 × 100 mm column, VanGuard guard column, and Sep‐Pak Vac C18 cartridges were obtained from Waters (Milford, Massachusetts), Glc_4_ from Toronto Research Chemicals (Toronto, Canada), glacial acetic acid, butyl‐4‐aminobenzoate, sodium cyanoborohydride from Sigma (St. Louis, MO), and methanol and acetonitrile (HPLC grade) from VWR Scientific products (Atlanta, Georgia). A stable isotope‐labeled Glc_4_ internal standard (IS) was synthesized as described.^24^

## 2 METHODS

### 2.1 Subjects

This was a single center, prospective study of patients consented to a natural history study, approved by Duke University Health System Institutional Review Board (#Pro00047556). Patients had a confirmed diagnosis of GSD III, via AGL variant and/or enzyme analysis (Supplemental Table 1).

### 2.2 Glucose tetrasaccharide and creatinine analyses

Glc_4_ was analyzed as a butyl‐4‐aminobenzoate derivative using [^13^C_6_]Glc_4_ as an IS, and ultraperformance liquid chromatograph‐tandem mass spectrometry (UPLC‐MS/MS), as reported with modifications.^24^ Urine (20 μL) was combined with 20 μL 50 μmol/L IS, incubated at 80°C for 1 hour with 152 mmol/L butyl‐4‐aminobenzoate, 400 mmol/L sodium cyanoborohydride, and 5.3% glacial acetic acid (vol/vol) in methanol, and excess reagent was removed using solid phase extraction. Samples were dried under nitrogen, reconstituted in 10 mmol/L ammonium acetate in 90:10 (vol:vol) acetonitrile: deionized water (diH_2_O), and separated with gradient elution on a UPLC BEH amide column using 10 mmol/L ammonium acetate in acetonitrile:diH_2_O as the mobile phase. Glc_4_ and the IS were detected by selected reaction monitoring (*m*/*z* 844 > 358 and *m*/*z* 850 > 364, respectively). Glc_4_ was normalized to creatinine (CN), analyzed as reported.^18^ The Glc_4_ assay has acceptable intraday and interday imprecision (≤20% over the calibration range: 2‐230 μmol/L).

### 2.3 24‐hour urine collections

Urine collections over 24 hours were conducted unsupervised in a residential setting. Subjects were instructed to discard the first morning void on day 1, and collect all subsequent voids separately over the next 24 hours, ending with the first morning void on the second day. Subjects were asked to reserve a small aliquot (about 1 mL) from each void in a separate container and combine the remaining urine in a single large container. Samples were stored in a cooler on cold packs and frozen within 48 hours of collection. Glc_4_ and creatinine were analyzed in each aliquot and pooled collections.

### 2.4 Statistical analyses

Descriptive statistics, Pearson correlation coefficient, linear regression, paired *t* test, and Bland‐Altman analyses were calculated using Microsoft Excel and GraphPad Prism V8. The diurnal variability of Glc_4_ excretion was calculated as the CV%. *P*‐values ≤.05 were considered significant.

## 3 RESULTS

### 3.1 Cohort description

Here, 18 subjects (n = 2 males, subjects 13 and 15) with GSD IIIa (n = 16) or b (n = 2, subjects 17 and 20) participated in the study. The median age was 1 year (range: 0.3‐12) at the time of diagnosis, and 13.5 years (range: 3.7‐62) at the start of the study. All were treated with various dietary regimens of cornstarch (Table 1), except the three adults (subjects #8, 13, and 29). The protein intake goal was 20% to 25% total energy consumed for all patients with GSD IIIa, achieved in seven subjects (#2, #5, #8, #10, #12, #13, and #17) using a protein supplement, and in the remaining subjects using natural sources of protein. Most subjects were ambulatory. One adult (#29) required a wheelchair and an 8‐year‐old female (#27) was considering a wheelchair for long distances. An 11‐year‐old male (#15) required ankle‐foot orthoses, an 8‐year‐old female (#7) was recommended to wear custom shoe inserts for calcaneal valgus, and one adult (#13) used assistive devices. Ten subjects had evidence of hepatomegaly on liver imaging. Of the three adults in the cohort, subject #8 showed evidence of liver fibrosis and cirrhosis, subject #13 underwent a multiorgan transplant (heart, liver, and kidney) after suffering heart failure,^25^ and subject #29 had HCC treated by radioembolization. The liver disease natural history in the pediatric subjects was reported in detail elsewhere.^20^

**TABLE 1:** Summary of diurnal variability of Glc_4_ excretion in subjects with GSD III

Subject ID‐collection #	Age (y)	Glc4 in 24‐h pooled urine (mmol/mol CN)	Diurnal variability (CV%)		Cornstarch dose and regimen					
					Daily dose (g/kg/d)	Breakfast	Lunch	Dinner	Before bed	Middle of night
			<25%	>25%		g/kg				
15‐1	10.8	1.7	16%		3.8	1.1			1.6	1.1
15‐2	11.8	2.2	4%							
15‐3	12.8	1.7		39%						
20‐1	13.5	2.0	14%		0.9				0.9	
20‐2	15.4	1.4	7%							
1‐1	14.6	6.6	NA		1.23	0.45			0.78	
08‐1	40.3	8.0	24%		None					
08‐2	40.7	10.4	10%							
08‐3	42.4	23.2	14%							
03‐1	15.1	8.8	7%		0.7				0.7	
03‐2	16.0	8.6	20%							
03‐3	17.1	16.7		47%						
18‐2	15.4	12.2	14%		0.73				0.73	
12‐1	3.7	11.6	12%		5.57	1.05	1.05	1.05	1.21	1.21
10‐1	4.9	9.5	25%		2.32	0.93			0.46	0.93
10‐2	7.2	4.4	12%							
09‐1	6.5	16.5	21%		1.9			1.1		0.8
09‐2	8.3	11.2		26%						
05‐1	9.6	16.8	10%		6	1.2	1.2	1.2	1.2	1.2
05‐2	11.3	14.3	5%							
27‐1	7.6	18.1		26%	3.12	0.78	0.78		1.56	
02‐1	13.9	20.2	8%		2.55	0.7	0.5	0.5	0.85	
17‐1	5.9	20.6	NA		2.29	0.38 every 4 h during the day, 0.76 by continuous feed at night				
14‐1	14.6	27.6		39%	0.64				0.64	
14‐2	16.7	28.0		30%						
13‐2	52.2	28.0	9%		None					
13‐3	53.2	25.7		28%						
29‐1	62.4	29.2	NA		None					
07‐1	8.2	42.2		39%	0.3				0.3	
19‐1	17.2	85.8		41%	0.63				0.63	
19‐2	19.1	33.6	12%							

*Note*: Collections by subjects 1, 17, and 29 were rejected for assessment of the diurnal variability, although pooled urine was used to determine Glc_4_ concentrations. All subjects have GSD IIIa except for subjects 17 and 20 who were diagnosed with GSD IIIb.

Abbreviations: GSD, glycogen storage disease; NA, not assessed.

### 3.2 24‐hour urine collections

Thirty‐six 24‐hour urine collections were completed by 18 subjects. Six subjects completed two and another six completed three repeat collections over 1 to 3 years. Then, 28 collections by 15 subjects were accepted for diurnal variability assessments (Table 1) and 8 collections (22%) were excluded due to incomplete or inaccurate collection or recorded information, or inappropriate storage. The total urine volume was positively correlated with age (Pearson *R*: .758, *P* < .05) and weight (Pearson *R*: .700, *P* < .05; Supplemental Figure 1). The median number of voids was 6 (range: 4‐10), the median total volume of urine collected was 1265 mL (range: 262‐4000), and the median urinary output was 1.0 mL/kg/h (range: 0.53‐2.7).

### 3.3 Cornstarch dose and Glc_4_ excretion

**FIGURE 1:**
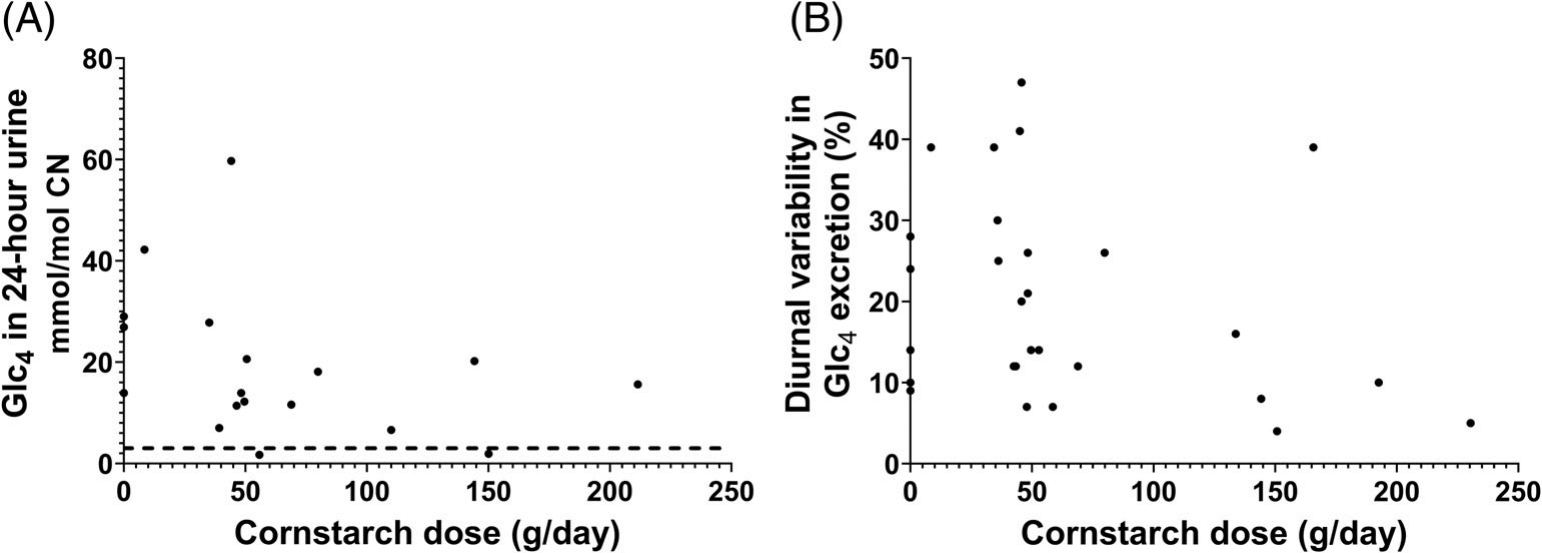
Comparison of the degree and variability of Glc_4_ excretion with uncooked cornstarch dose. A, Glc_4_ concentrations in 24‐hour urine relative to total cornstarch dose. No significant correlation was observed (Pearson *R* = −.349, *P* = .15, n = 18 patients). B, Variability (CV%) in Glc_4_ excretion in 24‐hour urine relative to cornstarch dose. No significant correlation was observed (Pearson *R* = −.278, *P* = .15, n = 28 collections by 15 subjects)

Glc_4_ was elevated in all subjects except an 11 year old subject with GSD IIIa (#15) and a 13 year subject old with GSD IIIb (#20) (Table 1). Glc_4_ concentrations normalized to creatinine in 24‐hour collections varied widely (median: 15 mmol/mol CN, range: 2‐60, n = 18; calculated using median values for subjects with more than one collection) and were not significantly correlated with the total cornstarch dose (Figure 1). Of note, both subjects with normal Glc_4_ (#15 and 20) were treated with cornstarch, whereas all three subjects (#8, 13, and 29) who were not on cornstarch therapy had elevated Glc_4_ (Table 1). For the 15 subjects with acceptable 24‐hour urine collections, the total amount of Glc_4_ excreted in 24 hours was significantly correlated with weight, but not age (Supplemental Figure 2). In comparison, the total amount of creatinine excreted over 24 hours significantly increased with weight and age (Supplemental Figure 2).

### 3.4 Variability in Glc_4_ excretion over 24 hours in patients with elevated Glc_4_

Glc_4_ was elevated in 23/28 of the 24‐hour urine collections, of which 15/23 had low (CV% ≤25), and 8/23 had higher (CV%: 26‐47) diurnal variability in Glc_4_ excretion (Table 1). Only 1/5 subjects who performed repeat collections (#14) had high variability in more than one collection. The diurnal variability was significantly correlated with the pooled 24‐hour urine Glc_4_ concentration normalized to creatinine (Pearson *R* = .644, *P* < .05), but not with the total dose of uncooked cornstarch (Figure 1). Furthermore, high variability was observed in one collection (#13‐2) from a subject not on cornstarch therapy. In contrast, low variability was observed in 11 collections by 8 subjects on cornstarch therapy.

No consistent trend in Glc_4_ excretion over 24 hours was observed in collections with high variability (Supplemental Figure 3). A closer agreement was observed between Glc_4_ concentrations in 24‐hour urines and the first void, compared with the last void collected (Supplemental Figure 4). However, the first and last voids did not differ significantly (paired *t* test, *P* = .41). For urines with low variability, both the first and last voids compared well with the 24‐hour urine (Supplemental Figure 5).

## 4 DISCUSSION

Glc_4_ is a promising biomarker in GSD III as it is correlated with serum transaminases in pediatric patients with GSD III^20^, ^23^ and a GSD III dog model,^26^ and with CK in adults with GSD III.^23^ These observations suggest urinary Glc_4_ reflects glycogen accumulation in liver more than muscle in the pediatric population, and muscle glycogen accumulation in adults. However, given the clinical variability of GSD IIIa and an increasing understanding of early muscle involvement, clinical correlation is needed in interpreting the source of Glc_4_ in GSD III. Patients with GSD III are treated with uncooked cornstarch which contains amylopectin, an α1‐6 branched glucose polymer that can be converted to Glc_4_ by amylase activity.^27^ This raises a concern for the reliability of using spot urines to assess Glc_4_ excretion in cornstarch‐treated patients. Our results suggest that uncooked cornstarch intake does not contribute significantly to Glc_4_ excretion.

A previous study investigating the impact of dietary factors on Glc_4_ reported diurnal variation in the rate of excretion in a volunteer on a normal diet over three days.^14^ There was evidence that the rate increased after meals; however, the overall daily excretion varied less than 10%. Decreased calorific intake and a low carbohydrate diet appeared to decrease the excretion rate, whereas a high carbohydrate diet consisting entirely of cooked rice (2400‐3200 kcal/24 h), resulted in a fourfold to fivefold rate increase, compared with a regular diet.^14^ The authors suggested this increase might be caused by amylopectin degradation by amylase in the gastrointestinal tract. Urinary output varies over the course of the day and is impacted by fluid intake, physical activity, and sleep.^28^ Thus, an increase in the Glc_4_ excretion rate under normal dietary conditions could be secondary, in part, to increased urinary output. The dose used to treat pediatric patients with GSD III is relatively low (typically 1 g/kg every 4 hours or longer, adjusted based on the ability of a dose to maintain euglycemia between feeds),^3^ and many adults with GSD III have a minimal intake of cornstarch. In comparison, patients with GSD I generally require higher and more frequent doses (eg, 1.6‐2.5 g/kg every 3‐5 hours) due to impairment in both glycogenolysis and gluconeogenesis.^29^ In our population, cornstarch intake ranged from 9 to 193 g/day, equivalent to approximately 30 to 700 kcal/day. This low dose and slower digestion of uncooked cornstarch compared with cooked starch, may explain the lack of correlation between cornstarch intake and the degree and variability of Glc_4_ excretion.

A higher variability in Glc_4_ excretion was associated with increased Glc_4_ concentrations, but was not consistently observed in repeat collections by the same subject. The reason for this variability is unknown. In addition to the potential for dietary influences, physical activity may be a factor.^14^ Twenty‐four hour urine collections are considered a “gold standard” method for assessing urinary biomarker excretion.^30^ However, 24‐hour collections impose a significant burden on subjects and a risk for collection errors,^31^ as demonstrated by the high rejection rate (22%) in our study. Appropriate storage of urine is another challenge. It was proposed that Glc_4_ might be unstable at ambient temperature in some urine samples because of bacterial degradation, resulting in specimens with unexpectedly low concentrations.^32^ Our studies indicate that Glc_4_ is usually stable in urine for 1 week in a climate‐controlled environment at ambient temperature (Supplemental Table 2). However, storage and transport of samples at 4°C or colder is recommended to ensure sample integrity. Several studies have demonstrated the equivalence of spot urines and 24‐hour collections for a number of analytes normalized to creatinine.^33^, ^34^, ^35^ Our results support the use of spot urines to evaluate Glc_4_ in patients with GSD III.

The reliance on patients to comply with the 24‐hour urine collection protocol and the prescribed dietary cornstarch therapy in an unsupervised setting was a limitation of this study. The reliability of the 24‐hour collections accepted for analysis is supported by several lines of evidence: (a) the Glc_4_ excretion rate (median: 45 mg/24 hours, range: 6‐720) was comparable to published rates in patients with GSD III (9‐45 mg/24 hours)^14^; (b) total urine volume and creatinine excreted over 24 hours significantly increased with age and weight; and (c) the voiding frequency and urine output were comparable to a previous study.^28^

To conclude, spot urines are generally reliable for assessing Glc_4_ excretion in GSD III. Increased variability in Glc_4_ excretion may be observed in patients who excrete higher Glc_4_ concentrations. This variability does not appear to be caused by cornstarch intake. Repeat assessments in spot urines collected close together in time are advisable, to assess baseline Glc_4_ concentrations and monitor trends in response to therapies in GSD III.

## CONFLICT OF INTEREST

S. P. Y. works for a laboratory that offers Glc_4_ testing, has received grant support from Sanofi Genzyme, Amicus Therapeutics, Biomarin Pharmaceutical, PTC Therapeutics, and Valerion Therapeutics, and has consulted for Amicus Therapeutics, Sanofi Genzyme, and PTC Therapeutics. P. S. K. has received research/grant support from Sanofi Genzyme, Valerion Therapeutics, Shire Pharmaceuticals, Amicus Therapeutics, Pfizer, Alexion Pharmaceuticals and Ultragenyx and consulting fees and honoraria from Sanofi Genzyme, Shire Pharmaceuticals, Alexion Pharmaceuticals, Amicus Therapeutics, Vertex Pharmaceuticals, Ultragenyx, and Asklepios Biopharmaceutical, Inc. (AskBio). P. S. K. is listed as an inventor on a licensed Duke University patent for the use of rhGAA in the treatment of GSDIII and other GSDs excluding GSDII. To date, neither Duke University nor the inventor has received any money from the commercialization of rights associated with this patent. S. A. has received consulting fees from Ultragenyx. The other authors declare no conflicts of interest.

## AUTHOR CONTRIBUTIONS

**Sarah P. Young**, **Stephanie Austin**, and **Priya S. Kishnani**: Contributed to the planning, conduct, and reporting of this work. **Ela Stefanescu** and **Andrea M. Seifts**: Contributed to the planning and conduct, and **Aleena Khan** and **Ghada Hijazi**: Contributed to the reporting of this work.

## INFORMED CONSENT

All procedures followed were in accordance with the ethical standards of the responsible committee on human experimentation (institutional and national) and with the Helsinki Declaration of 1975, as revised in 2000 (5). Informed consent was obtained from all patients for being included in the study.

## Supporting information


**Appendix** **S1:** Supplementary Information
