# Supplementary material for: Diurnal variability of glucose tetrasaccharide (Glc4) excretion in patients with glycogen storage disease type III
Source: JIMD Rep. 2020 Nov 3;58(1):37–43. doi: 10.1002/jmd2.12181 (PMC7932871; doi:10.1002/jmd2.12181)
Supplement: Supplementary file 1 — Appendix S1: Supplementary Information [file JMD2-58-37-s001.docx]

Supplemental table 1: *AGL* Variants

| **Subject** | **Variant** | **Nucleotide Change** | **Amino Acid Change** | **^d^Exon** | **^d^Intron** | **ClinVar ID** | **ClinVar Classification** |
| --- | --- | --- | --- | --- | --- | --- | --- |
| 1 | Variant 1 | c.2309-1G>A |  |  | 17 | 188804 | P/ LP​ |
|  | Variant 2 | c.4260-12A>G |  |  | 31 | 1099 | P/ LP​​ |
| 3 | Variant 1 | c.2309-1G>A |  |  | 17 | as above | as above |
|  | Variant 2 | c.4260-12A>G |  |  | 31 | as above | as above |
| 5^a^ | Variant 3 | c.1276del | p.Val426fs | 10 |  | None |  |
|  |  | Not found |  |  |  |  |  |
| 7 | Variant 4 | c.3259+3A>T |  |  | 24 | 553150 | VUS |
|  | Variant 4 | c.3259+3A>T |  |  | 24 | as above | as above |
| 8 | Variant 5 | c.966dup68 |  | 8 |  | None |  |
|  | Variant 6 | c.2590C>T | p.Arg864Ter | 20 |  | 21215 | P |
|  | Variant 7 | c.4529dup | p.Tyr1510Ter | 34 |  | 1094 | P/ LP​ |
| 9^b^ | Variant 8 | c.3299del | p.Gly1100fs | 25 |  | None |  |
|  | Variant 9 | c.3854A>C | p.Glu1285Gln | 29 |  | None |  |
| 10 | Variant 10 | c.2929C>T | p.Arg977Ter | 22 |  | 370992 | P/ LP​​ |
|  | Variant 11 | c.3866T>C | p.leu1289pro | 29 |  |  |  |
| 12 | Variant 12 | c.4221dup | p.Leu1408fs | 31 |  | 189052 | P/ LP​​ |
|  | Variant 2 | c.4260-12A>G |  |  | 31 | as above | as above |
| 13 | Variant 13 | c.100C>T | p.Arg34Ter | 3 |  | 188743 | P/ LP​​ |
|  | Variant 6 | c.2590C>T | p.Arg864Ter | 20 |  | as above | as above |
| 14 | Variant 14 | c.1384del | p.Trp461_Val462insTer | 11 |  | 189080 | P/ LP​ |
|  | Variant 14 | c.1384del | p.Trp461_Val462insTer | 11 |  | as above | as above |
| 15 | Variant 15 | c.2023C>T | p.Arg675Trp | 16 |  | 631558 | VUS |
|  | Variant 16 | c.3980G>A | p.Trp1327Ter | 30 |  | 1108 | P |
| 17^c^ | Variant 17 | c.18_19del | p.Gln6fs | 2 |  | 195097 | P/ LP​ |
|  | Variant 18 | c.293+1del |  |  | 3 | 370565 | LP |
| 18 | Variant 19 | c.2681+1G>T |  |  | 20 | 371344 | P |
|  | Variant 19 | c.2681+1G>T |  |  | 20 | as above | as above |
| 19 | Variant 19 | c.2681+1G>T |  |  | 20 | as above | as above |
|  | Variant 19 | c.2681+1G>T |  |  | 20 | as above | as above |
| 20^c^ | Variant 17 | c.18_19del | p.Gln6fs | 2 |  | as above | as above |
|  | Variant 20 | c.757G>C | p.Ala253Pro | 6 |  | None |  |
| 27 | Variant 16 | c.3980G>A | p.Trp1327Ter | 30 |  | as above | as above |
|  | Variant 21 | c.4259+1G>A |  |  | 31 | None |  |

VUS: Variant of Uncertain Significance, LP: Likely Pathogenic, P: Pathogenic

Variants referenced using transcript NM_000642.3(AGL).

*AGL* testing results was not available for subjects 2 and 29.

^a^ Only one variant detected. Supporting diagnostic information included the clinical presentation and low GDE activity in liver.

^b^ Subject 9 is homozygous for c.3299del, and has an third variant, c.3854A>C, in *AGL*

^c^ Variant c.18_19del is associated with the GSD IIIb phenotype. This variant was previously assigned to exon 3, and is now assigned to exon 2, according to a RefSeqGene GRCh38.p13

^d^ Exon and intron numbers are listed according to GRCh38.p13

**Supplemental Figure 1: Total urine volume in 24-hour collections relative to age and weight**

Total 24-urine output was significantly correlated with age and weight (n = 14). Each subject is represented once. Total volumes not available for 4 subjects.

1. Correlation of total urine volume with age: Pearson R: 0.843, p<0.05 Y = 70*X + 333
2. Correlation of total urine volume with weight: Pearson R: 0.811, p<0.05; Y = 42*X - 483

**Supplemental Figure 2: Correlation of the total Glc_4_ and creatinine excreted over 24 hours with age and weight.**

The total Glc_4_ excreted in 24 hours was significantly correlated with weight, but not age (n=14). Creatinine excreted in 24 hours was significantly correlated with both weight and age (n = 14).

1. Glc_4_  vs age, r = 0.291, p=0.31
2. Glc_4_  vs weight, r = 0.533, p=0.05
3. Creatinine vs age, r = 0.724, p<0.05
4. Creatinine vs weight, r = 0.824, p<0.05

**Supplemental Fig 3:** Trends in Glc_4_ excretion over 24 hours for eight collections that had high (>25%) variability in Glc_4_ excretion

After discarding the first morning urine on Day 1, subjects were instructed to collect all other voids on Day 1 and the first void on Day 2. Designating the first collected void on Day 1 as “0 hours”, Glc_4_ normalized to creatinine were plotted against time.

**Supplemental Fig 4:** First and last voids vs. 24-hour urine in high variability collections with elevated Glc_4_

Linear regression and Bland-Altman analysis of Glc_4_ concentrations in the first (time 0) or last (first-morning void on day 2) voids with the 24-hour urine Glc_4_ in collections with high (>25%) variability. 95% confidence intervals are shown in parentheses. (n = 8).

1. First void vs. pooled 24-hour collection.

y = 1.2 (0.36-2.1)x -4.8 (-26-17), R^2^ = 0.67, p<0.05;

1. Last vs. pooled 24-hour collection

y = 0.62 (0.34-0.89)x + 5.2 (-1.6-12), R^2^ = 0.84, p<0.05.

1. Bland-Altman analysis of first void vs. pooled 24-hour collection

Mean bias (SD): 0.31 (9.3); 95% limits of agreement: -18 to 19

1. Bland-Altman analysis of last void vs. pooled 24-hour collection

Mean bias (SD): -3.5 (4.9); 95% limits of agreement: -13 to 6.1

**Supplemental Fig 5:** First and last voids vs. 24-hour urine in low variability collections with elevated Glc_4_

Linear regression and Bland-Altman analysis of Glc_4_ concentrations in the first (time 0) or last (first-morning void on day 2) voids with the 24-hour urine Glc_4_ in collections with low (≤25%) variability. 95% confidence intervals are shown in parentheses. (n = 15).

1. First void vs. pooled 24-hour collection.

y = 0.92 (0.83-1.0)x -0.49 (-0.94-1.9), R^2^ = 0.96, p<0.05;

1. Last vs. pooled 24-hour collection.

y = 0.92 (0.79-1.0)x + 0.5 (-1.3 – 2.3), R^2^ = 0.93; p<0.05.

1. Bland-Altman analysis of first void vs pooled 24-hour collection

Mean bias (SD): -0.48 (2.1); 95% limits of agreement: -4.6 to 3.7

1. Bland-Altman analysis of last void vs pooled 24-hour collection

Mean bias (SD): -0.60 (2.7); 95% limits of agreement: -5.8 to 4.6

Supplemental Table 2: Stability of Glc_4_ at ambient temperature

| Days in storage | Sample 1 | | Sample 2 | |
| --- | --- | --- | --- | --- |
|  | Conc | % change from baseline | Conc | % change from baseline |
|  | µmol/L |  | µmol/L |  |
| 0 | 41 |  | 156 |  |
| 1 | 40 | -2% | 165 | 6% |
| 2 | 37 | -10% | 159 | 2% |
| 4 | 35 | -15% | 146 | -6% |
| 5 | 33 | -20% | 156 | 0% |
| 6 | 44 | 7% | 189 | 21% |
| 8 | 39 | -5% | 145 | -7% |
| 13 | 34 | -17% | 126 | -19% |

Samples with low and high concentrations of Glc_4_ were stored at ambient room temperture for up to 13 days. These data suggest Glc_4_ is stable for at least 8 days under these conditions.
